# Are Extremes of Consumption in Eating Disorders Related to an Altered Balance between Reward and Inhibition?

**DOI:** 10.3389/fnbeh.2014.00410

**Published:** 2014-12-09

**Authors:** Christina E. Wierenga, Alice Ely, Amanda Bischoff-Grethe, Ursula F. Bailer, Alan N. Simmons, Walter H. Kaye

**Affiliations:** ^1^Department of Psychiatry, University of California San Diego, La Jolla, CA, USA; ^2^Veterans Affairs San Diego Healthcare System, San Diego, CA, USA; ^3^Department of Psychiatry and Psychotherapy, Division of Biological Psychiatry, Austria Medical University of Vienna, Vienna, Austria

**Keywords:** anorexia nervosa, bulimia nervosa, eating disorders, reward processing, inhibition, cognitive control, gustatory processing

## Abstract

The primary defining characteristic of a diagnosis of an eating disorder (ED) is the “disturbance of eating or eating-related behavior that results in the altered consumption or absorption of food” (DSM V; American Psychiatric Association, 2013). There is a spectrum, ranging from those who severely restrict eating and become emaciated on one end to those who binge and overconsume, usually accompanied by some form of compensatory behaviors, on the other. How can we understand reasons for such extremes of food consummatory behaviors? Recent work on obesity and substance use disorders has identified behaviors and neural pathways that play a powerful role in human consummatory behaviors. That is, corticostriatal limbic and dorsal cognitive neural circuitry can make drugs and food rewarding, but also engage self-control mechanisms that may inhibit their use. Importantly, there is considerable evidence that alterations of these systems also occur in ED. This paper explores the hypothesis that an altered balance of reward and inhibition contributes to altered extremes of response to salient stimuli, such as food. We will review recent studies that show altered sensitivity to reward and punishment in ED, with evidence of altered activity in corticostriatal and insula processes with respect to monetary gains or losses, and tastes of palatable foods. We will also discuss evidence for a spectrum of extremes of inhibition and dysregulation behaviors in ED supported by studies suggesting that this is related to top-down self-control mechanisms. The lack of a mechanistic understanding of ED has thwarted efforts for evidence-based approaches to develop interventions. Understanding how ED behavior is encoded in neural circuits would provide a foundation for developing more specific and effective treatment approaches.

## Clinical Presentation of Anorexia Nervosa and Bulimia Nervosa

Individuals with eating disorders (ED) have puzzling symptoms that are unique to the disorder, such as dysregulated eating (restricting, binging/purging), relentless drive to lose weight, body image distortions, and denial of illness. The primary defining characteristic of ED is abnormal extremes of food consumption. Some individuals lose weight only by severely restricted dieting [e.g., anorexia nervosa (AN)], often becoming emaciated, while others alternate between restricting and episodic binge eating and/or purging [e.g., bulimia nervosa (BN)]. However, many individuals with an ED do not have a “pure” diagnosis of AN or BN. Moreover, it is common for individuals to change subtypes over time, or have a mixture of symptoms (e.g., AN and BN). Advances in our understanding of the neurocircuitry of ED and how such symptoms are encoded in the brain are beginning to raise the possibility that EDs may be better understood based on a continuum of interacting neurocognitive constructs underlying consummatory behavior. Simply put, we propose that extremes of consumption in ED are related to an altered balance between reward and inhibition. While a relatively novel idea for understanding ED, this perspective is consistent with the obesity and addiction literature. These fields have made substantial advances in delineating the circuitry that makes drugs and food rewarding, as well as the self-control mechanisms that may inhibit their use, which may serve as models to develop a mechanistic understanding of ED. Revealing brain mechanisms underlying ED pathophysiology will not only advance our understanding of the neurobiology underlying the puzzling symptoms of ED, but importantly, help guide disease-specific treatment development strategies.

## Factors That are Shared Across ED Subtypes

Although psychosocial factors have been hypothesized to cause ED, recent studies show that genetic heritability accounts for approximately 50–80% of the risk and creates neurobiological vulnerabilities (Kendler et al., 1991; Berrettini, 2000; Bulik et al., 2006; Kaye et al., 2008). It has been argued that individuals with ED share some common risk and liability factors because these disorders are often cross-transmitted in families (Kendler et al., 1991; Walters and Kendler, 1995; Lilenfeld et al., 1998; Strober et al., 2000). Importantly, individuals with an ED tend to share certain temperament and personality traits, which often first occur in childhood before the onset of an ED and may create or increase a vulnerability to develop an ED. For example, individuals with an ED tend to have difficulties with emotional regulation and negative affect (Lavender et al., under review), characterized by exaggerated trait anxiety (Lilenfeld et al., 2006), high incidence of co-morbid anxiety disorders (Lilenfeld et al., 2006), elevated intolerance of uncertainty (Frank et al., 2012a), and exaggerated harm avoidance, a temperament trait (Cloninger et al., 1994) that contains elements of anxiety, inhibition, and inflexibility (Fassino et al., 2004; Klump et al., 2004; Cassin and von Ranson, 2005; Wagner et al., 2006; Lilenfeld, 2011). They also share traits including perfectionism (Friederich and Herzog, 2011), obsessionality (Anderluh et al., 2003; van den Heuvel et al., 2005), interoceptive deficits (Lilenfeld et al., 2006), and increased sensitivity to punishment (Harrison et al., 2010; Jappe et al., 2011). These temperament and personality factors persist after recovery, further elevating the likelihood that these are stable traits that persist beyond remittance of pathologic consumptive behaviors (Wagner et al., 2006). Individuals with AN and BN report global difficulties with effectively regulating their emotional experiences (Brockmeyer et al., 2013), and ED behaviors tend to be used for coping with or modulating emotion. In AN, dietary restraint and reduced daily caloric intake appears to serve as a means of anxiety reduction (Vitousek and Manke, 1994; Kaye et al., 2003; Steinglass et al., 2010), whereas eating stimulates dysphoric mood (Frank and Kaye, 2012). For BN, while negative affect, mood lability, and stress may trigger binge-eating episodes, the binge and purge cycle may in fact reduce dysphoria and/or anxiety (Abraham and Beaumont, 1982; Johnson and Larson, 1982; Kaye et al., 1986; Smyth et al., 2007; Crosby et al., 2009; Haedt-Matt and Keel, 2011). As we discuss below, an enhanced sensitivity to anxiety and punishment may contribute to a shared temperament characterized by dysregulated valuation of reward.

## Factors That Differentiate ED Subtypes

It is well known that individuals with AN and BN tend to have opposite extremes of inhibitory self-control. Behavioral inhibition is characterized by overcontrol, passivity, and constricted affect/thinking (Claes et al., 2006; Lilenfeld et al., 2006; Marsh et al., 2009). Pure restrictor-type AN individuals (Kaye et al., 2009) tend to be over-controlled and over-concerned about consequences, whereas those with BN and AN-BN tend to have poor impulse control and are less paralyzed by concerns with future consequences (Wu et al., 2013). For example, ill adults with AN demonstrate an enhanced ability to delay monetary reward (Steinglass et al., 2012), though this behavior appears to normalize with weight restoration (Steinglass et al., 2014) and remittance of symptoms (Wierenga et al., 2014a). This ability to delay reward, which may reflect reduced reward sensitivity, increased cognitive control, or both, may help to maintain persistent food restriction. In contrast, individuals with BN and AN-BN tend to have poor impulse control, and engage in greater novelty, pleasure, and stimuli seeking behavior (Strober et al., 1997; Wagner et al., 2006). This dysregulation parallels their eating patterns, and may contribute to the primary symptoms in BN of binge eating and compensatory behaviors.

Previous studies from our group are consistent with this conceptualization. In 2006, we employed a latent class analysis (Wagner et al., 2006) of temperament, behavioral, and symptom self-report assessments in 55 women recovered (REC) from an ED (i.e., AN, AN-BN, and BN), and two clusters emerged. Women in one cluster were more inhibited, with high levels of harm avoidance and state anxiety. Women in the other cluster were dysregulated and were characterized by greater impulsivity but also had some degree of anxiety and obsessionality. This demonstrates that individuals have similar inhibited and dysregulated temperament and personality factors when ill and recovered. Notably, the clusters were not related to DSM diagnosis, which mirrors other classification studies (Clinton et al., 2004; Sloan et al., 2005; Krug et al., 2011) suggesting alternate classification schemes may be more closely associated with ED etiology.

## Proposed Model to Explain Extremes of Food Consumption in Eating Disorders

A small but relatively consistent literature tends to show that reward and inhibition are core dimensions in ED (Goldner et al., 1999; Westen and Harnden-Fischer, 2001; Karwautz et al., 2003; Wonderlich et al., 2005; Claes et al., 2006; Holliday et al., 2006; Thompson-Brenner et al., 2008; Peñas-Lledó et al., 2010; Gazzillo et al., 2013). Our *overarching hypotheses* is that AN, AN-BN, and BN share a disturbance of valuation of reward, and that this altered reward appraisal may be related to emotional dysregulation, manifesting as exaggerated anxiety and sensitivity to punishment. In contrast, inhibitory function varies between diagnoses, and ED may represent a spectrum anchored by extremes of inhibition and dysregulation. Evidence suggests individuals with AN may compensate for dysfunctional reward processing using exaggerated cognitive control. In contrast, those with BN may have deficient cognitive control, thus increasing instability and erratic responding to appetitive stimuli. According to this model, extremes of eating behavior emerge from an altered balance of reward and inhibitory processing. We discuss the behavioral, cognitive, and neuroimaging evidence that give rise to this model. We provide an overview of the neurocircuitry supporting reward and inhibition and review what is known about these processes in ED and how they differ amongst subtypes. Lastly, we discuss the clinical implications of our model and how this conceptualization might be applied to treatment.

## Neurocircuitry Modulating Reward and Inhibition

As described by Phillips et al. (2003), ventral limbic neural circuitry, which includes the rostral anterior cingulate cortex (ACC), ventromedial prefrontal cortex (PFC), and anterior ventral striatum (a functional subdivision comprising the nucleus accumbens, rostroventral putamen, and ventromedial caudate) (Haber and Knutson, 2010), is necessary for identifying and valuating rewarding or emotionally significant stimuli and for generating affective responses to these stimuli (Delgado et al., 2000; McClure et al., 2004, 2007; Wittmann et al., 2007; Haber and Knutson, 2010; Wittman et al., 2010; Kim et al., 2011; Onoda et al., 2011; Sripada et al., 2011). A second system, supporting cognitive control, inhibition, planning, effortful regulation of affective states, and decision-making, is modulated by a dorsal cognitive neural circuit, which includes the dorsal caudate, dorsal ACC (dACC), ventrolateral and dorsolateral PFC (DLPFC), insula, and parietal cortex (McClure et al., 2004, 2007; Wittmann et al., 2007; Wittman et al., 2010). As part of the cognitive circuit, the dACC, which has extensive reciprocal connections with the DLPFC (Dietz, 1998) and the dorsal caudate (Haber and Knutson, 2010), monitors behavior in potential conflict situations (Paus et al., 2001; Walton et al., 2003; Vogt et al., 2005; Dixon et al., 2006). Given their anatomical connections, the dACC and dorsal caudate in particular may work together in considering prior outcomes when making choices. A third system, part of the salience network, centers on the anterior insula, which is a hub for the evaluation of interoceptive cues, such as pain, tastes, or feelings of fullness, and for integrating these cues with motivational and emotional processes to give rise to conscious visceral perception of homeostatic states through the involvement of subcortical homeostatic control centers (i.e., hypothalamus, amygdala) and cortical control centers (i.e., DLPFC) (Craig, 2002, 2004, 2009). Overall, these limbic, cognitive, and salience circuits interact to valuate reward, assess future consequences of one’s behavior, and integrate and evaluate reward prediction to guide decisions using cognitive control and inhibition.

Dysfunction within these regions has been proposed to be a key neural mechanism underlying altered behavioral regulation, reward regulation, or cognition found in addiction (Goldstein and Volkow, 2002; Feil et al., 2010). The addiction and obesity literature has made substantial advances in understanding the neural circuitry involved in encoding the salience and regulating the rewarding effects of food and drugs, as well as the corresponding behaviors of approach and avoidance (Bruce et al., 2011; Burger and Stice, 2011; Carnell et al., 2012; Volkow et al., 2012). In contrast, less is known about this neural circuitry and its relationship to dysregulated eating behavior in ED. Still, there is evidence (discussed below) that disturbances of such circuitry occur in ED, persist after recovery (Kaye et al., 2013), and play a critical role in a vulnerability for altered eating behavior. Application of concepts (Bruce et al., 2011; Burger and Stice, 2011; Carnell et al., 2012; Volkow et al., 2012) that have been used in the study of obesity and addiction may further define these neural networks and their relationship to behavior in ED. This approach also holds the promise of providing the information needed to understand how ED psychopathology may be related to a larger spectrum of disorders of abnormal consummatory behavior.

## Reward Processing in EDs

### Behavioral evidence of altered reward sensitivity in ED

Individuals with clinical and subclinical EDs have high punishment sensitivity in the ill and recovered states (Claes et al., 2006; Harrison et al., 2010, 2011; Jappe et al., 2011; Matton et al., 2013; Glashouwer et al., 2014), with higher punishment sensitivity in purging subtypes (Glashouwer et al., 2014). This may explain why ED individuals tend to perceive their actions as incorrect or flawed and are highly sensitive to criticism; this bias likely interferes with motivation or ability to learn from experience. Individuals with BN tend to have increased sensitivity to reward (SR) (Harrison et al., 2010; Chan et al., 2014) but findings are mixed in AN. Some studies report decreased reward sensitivity in restricting-type AN but increased reward sensitivity in purging type AN (Claes et al., 2006; Harrison et al., 2010), while others report increased SR in both AN subtypes (Jappe et al., 2011; Glashouwer et al., 2014). This discrepancy may be due to differences in behavioral measures used, as studies that showed lower reward sensitivity in AN tend to use “fun seeking” (Carver and White, 1994) or novelty seeking measures (Cloninger et al., 1994), which assess impulsivity (Franken et al., 2006). In contrast, studies that use the Sensitivity to Punishment and Sensitivity to Reward Questionnaire (SPSRQ) (Torrubia et al., 2001) tend to show increased reward sensitivity across all ED subtypes, thought to be driven by endorsement of items specific to increased sensitivity to appearance and interpersonal reward. In summary, evidence suggests alteration in the valuation of rewarding and aversive stimuli is a transdiagnostic temperament trait that plays a role in the development and maintenance of ED.

### Neuroimaging evidence of altered response to monetary reward in ED

Animal studies show that the ventral striatum processes motivational aspects of stimuli by modulating the influence of limbic inputs on striatal activity (Schultz, 2004; Yin and Knowlton, 2006). In this way, even secondary rewards such as money activate the ventral striatum proportionally to the reward amount or deviation from an expected payoff (Montague et al., 2004). Typically, healthy, normal weight human beings tend to have a more robust response to positive relative to negative feedback within the ventral striatum and dorsal caudate, suggesting they are more sensitive to reward than punishment (Delgado et al., 2000). A different pattern is emerging in studies of ED. Using a monetary choice task (Delgado et al., 2000), ill AN adolescents (Bischoff-Grethe et al., 2013), REC AN (Wagner et al., 2007), and REC BN (Wagner et al., 2010) failed to differentiate feedback valence (wins and losses) in ventral-striatal regions in comparison to controls, suggesting difficulty in discriminating positive and negative feedback, perhaps related to increased sensitivity to both reward and punishment. Instead, REC AN participants showed an exaggerated response to both reward and punishment in dorsal executive regions (e.g., dorsal caudate, DLPFC, parietal cortex) relative to healthy peers, pointing to enhanced inhibitory activity, which may be linked to anxiety, given positive correlations between anxiety and brain response in the caudate in REC AN (Wagner et al., 2007). While the magnitude of dorsal caudate response was normal in REC BN (Wagner et al., 2010), it failed to distinguish responses to positive and negative feedback. As the dorsal caudate has been associated with inhibitory control (Konishi et al., 1998), one possible interpretation is that REC BN have disturbed executive function in the linking of stimulus cues with valenced outcomes, reflected in their occasionally impulsive, disinhibited behavior. Ill AN adolescents also exhibited an exaggerated response to losses compared to wins in posterior executive and sensorimotor striatal regions (Bischoff-Grethe et al., 2013). Together, these data support the hypothesis that AN and BN experience altered reward sensitivity related to limbic (reward) circuitry but differ in their inhibitory response, with increased behavioral inhibition related to overactive cognitive neural circuitry in AN and decreased inhibitory control as manifested in dysfunctional executive processes in BN (Dietz, 1998; Phillips et al., 2003).

To further examine whether diminished response to reward could underlie food restriction in AN, we investigated brain activation during delay discounting in REC AN when hungry and when satiated, given that hunger influences behavioral choice in healthy individuals by increasing the appetitiveness of rewarding stimuli (Goldstone et al., 2009; Wang and Dvorak, 2010; Levy et al., 2013; Tal and Wansink, 2013). Compared to comparison women, REC AN failed to increase activation of reward valuation circuitry when hungry and showed elevated response in cognitive control circuitry regardless of metabolic state (Wierenga et al., 2014a). This finding is consistent with our previous studies (Wagner et al., 2007, 2010; Bischoff-Grethe et al., 2013) and others showing limbic regions are underactive for motivational behavior in ill AN (Zastrow et al., 2009). The lack of susceptibility to hunger-driven, reward-seeking behavior raises the possibility that this pathophysiology may play a critical role in the successful food restriction characteristic of AN, in that hunger does not make salient stimuli more appetitive in REC AN. Moreover, difficulties in valuating emotional salience may contribute to inabilities to appreciate the risks inherent in this deadly disorder.

### Neuroimaging evidence of altered response to food reward in ED

Studies of secondary rewards such as money have the benefit of examining reward processing without the possible confound of symptom provocation, such as anxiety or body image distortions that food-related stimuli may elicit in individuals with an ED. However, brain response to food-related stimuli may elucidate reward processing that is more directly related to eating pathology. Visual processing of images of food is thought to activate anticipatory responses that determine future feeding behavior (Stice et al., 2009). Studies that examine brain response to images of food tend to report reduced activation in the insula, lateral PFC, and parietal lobe with increased medial PFC activation in adults with AN (Uher et al., 2003; Gizewski et al., 2010; Holsen et al., 2012; Kim et al., 2012), and increased insula activation in adults with BN (Schienle et al., 2009; Brooks et al., 2011; Weygandt et al., 2012) (see Garcia-Garcia for review) (Garcia-Garcia et al., 2013) implicating both cognitive and reward circuitry. However, studies that manipulate the relationship of food cues to food receipt are better positioned to examine response to anticipation and consumption of primary reward, and may provide unique information about primary reward processing in ED. For example, biobehavioral research highlights the importance of distinguishing between consummatory food reward and the anticipation of that reward in obesity (Stice et al., 2008) and addiction (Volkow et al., 2012). Of potential relevance to ED, studies (Epstein et al., 2004, 2007) suggest that the reward anticipated from food is a more robust determinant of intake than the reward experienced when the food is actually consumed. Animal studies (Stice et al., 2008) have shown that stimulus conditioning leads to a shift in the firing of dopamine (DA) neurons from food consumption to the cues associated with consumption, or the anticipation of food reward, wherein cues linked with food receipt begin to elicit anticipatory food responses.

Functional neuroimaging studies have distinguished neurocircuitry modulating the anticipatory phase and the outcome phase in salience and incentive processing (Grupe and Nitschke, 2013). The insula plays a pivotal role in anticipation and processing of interoceptive states, including taste, by conveying information about the internal milieu of the organism via the spino-thalamo-cortical interoceptive pathway (Craig, 2002). Through primary representation of the sensory state in the posterior insula, this information gets re-represented within the mid-insula through integration with subcortical homeostatic control centers (i.e., hypothalamus, amygdala), which is further re-represented within the anterior insula through integration with the cortical control regions (i.e., DLPFC). The anterior insula thus appears to be a hub for the integration and evaluation of interoceptive cues, such as tastes or feelings of fullness, with motivational and emotional processes to give rise to conscious visceral perception of homeostatic states (Craig, 2002, 2004, 2009). That is, the insula is thought to code interoceptive prediction error, signaling mismatch between actual and anticipated bodily arousal, which in turn elicits subjective anxiety and avoidance behavior (Craig, 2002, 2009; Paulus and Stein, 2006). Brain imaging studies giving tastes of sugar to healthy individuals in food-deprived compared with satiated states have consistently shown that receipt of sucrose in the food deprivation state results in relatively higher activation in the insula and orbitofrontal cortex, potentially reflecting increased sensitivity to change in interoceptive state (Haase et al., 2009).

A growing body of research identifying correlates of anticipation and receipt of food reward in AN (Kaye et al., 2013) suggests that ill and REC AN adults have increased anticipatory response in the anterior insula, striatum, and frontal regions to food cues (Cowdrey et al., 2011; Frank et al., 2012b; Oberndorfer et al., 2013a,b), and reduced response in the anterior insula and striatum to tastes of palatable foods (Wagner et al., 2008; Vocks et al., 2011; Oberndorfer et al., 2013a). Specifically, our group has found that REC AN adults demonstrate increased insula response to cues (Oberndorfer et al., 2013b) and reduced insula response when tasting sucrose (Oberndorfer et al., 2013a). Frank et al. (2012b) found that, compared to comparison women, neural circuits associated with reward learning were more active in ill AN adults in the anterior ventral striatum, insula, and orbitofrontal cortex when engaging in an associative learning task of conditioned visual stimuli and sucrose taste, suggesting enhanced activation to unexpected taste stimuli in reward circuitry in AN. Further, in a complex paradigm combining anticipation and receipt of reward through viewing and tasting randomly presented pleasant and unpleasant stimuli (Cowdrey et al., 2011), REC AN demonstrated increased activity in the ventral striatum in response to both sights and flavors of pleasant stimuli (chocolate) and increased activity in the insula and posterior dorsal caudate to both flavors and sights of aversive stimuli when compared to controls. An exaggerated response to stimulus cues may be a means to predict and manage the anxiety elicited by subjectively aversive stimuli, similar to the anticipatory sensitivity linked with stimulus avoidance that is seen in highly anxious individuals (Simmons et al., 2006). Exaggerated response in reward circuitry to unexpected taste receipt may reflect altered DA-mediated reward processing, as unexpected rather than predictable stimulation is related to DA activation [Schultz (2002); see Kaye et al. (2013) for discussion of the role of DA in AN]. Taken together, AN individuals able to restrict their eating appear to have an enhanced anxiety response to anticipated food cues, and diminished insula and striatal response to receipt of food.

In contrast, individuals with BN appear to demonstrate the opposite processing pattern. Ill BN individuals have reduced anticipatory response to food cues in the insula and striatum (Bohon and Stice, 2011; Frank et al., 2011) and increased response to receipt of food in the insula and striatal regions (Radeloff et al., 2012; Oberndorfer et al., 2013a). Frank et al. (2011) found that ill BN individuals showed reduced brain response to unexpected reward receipt or omission of taste stimuli in a paradigm in which conditioned visual stimuli were linked with unconditioned sucrose taste. These ill BN individuals also demonstrated reduced brain response to a DA-related reward learning task in the insula, ventral putamen, amygdala, and orbitofrontal cortex. These studies suggest that in contrast to AN, BN may have deficits in reward responding to food cues, which may in turn contribute to overeating or binge-eating behavior. Animal studies suggest that while food restriction may sensitize, excessive food intake may desensitize brain reward pathways (Johnson and Kenny, 2010; Avena et al., 2012). For example, obese individuals show blunted reward-based activation to food receipt, but greater activation in regions that encode the reward value of food cues (Stice et al., 2011). Overeating, and by extension binge eating, leads to reduced striatal response to food receipt and greater responsivity to conditioned food cues. The research showing reduced anticipatory and enhanced consummatory response to food reward in BN suggests that this pattern of responding may serve to maintain the binge-eating behavior characteristic of the disorder. Considered together, data in AN and BN suggest that individuals with an ED have altered or dysfunctional sensitivity for sensory-interoceptive-reward processes when consuming palatable foods (Small, 2009). While undereaters’ baseline rate of responding may mimic a continuous state of satiety, limiting interoceptive and reward processing, overeaters may have chronically hyper-active brain circuits, manifesting as a state of subjective deprivation. Evidence of reduced structural brain volume of the striatum in women with AN or BN, which was shown to be associated with SR, provides further support for altered brain reward circuitry in AN and BN (Frank, 2013).

## Inhibitory Cognitive Control in ED

### Behavioral evidence of altered inhibitory cognitive control in ED

Individuals with pathologic eating have dysregulated inhibitory control and tend to be overly inhibited or impulsive (Cassin and von Ranson, 2005; Frank et al., 2005; Claes et al., 2006; Lilenfeld et al., 2006; Wagner et al., 2006; Marsh et al., 2009; Steinglass et al., 2012). Inhibitory cognitive control is a critical executive function involved in regulating behavior and emotions (Harnishfeger and Pope, 1996). An impaired ability to overcome inhibition or switch behaviors may underlie symptoms in people with ED (Holliday et al., 2005; Schmidt and Treasure, 2006). For example, underweight ill AN adults are impaired in cognitive set-shifting (Beaton et al., 1979; Tchanturia et al., 2002, 2012; Steinglass et al., 2006; Roberts et al., 2007; Nakazato et al., 2009, 2010; Friederich and Herzog, 2011; Stedal et al., 2012), as evidenced by elevated perseverative errors, but perform better than binging/purging ED subtypes on tests of cognitive switching (e.g., Trail Making Test) and inhibition (e.g., Stroop task) (Claes et al., 2010, 2012). Individuals with binge/purge subtypes report less behavioral inhibition than restrictive subtypes on the Effortful Control Scale (Evans and Rothbart, 2007; Claes et al., 2010), and the level of inhibition is negatively correlated with better performance on the Stroop task (Claes et al., 2010). Individuals with BN and AN binge-purge subtype also demonstrate higher motoric impulsivity than restricting-type AN on the Barratt Impulsivity Scale (Rosval et al., 2006). A recent meta-analysis (Wu et al., 2013) that identified 24 studies of neurocognitive inhibition in bulimic-type ED found that individuals in these groups (BN, AN-BN, or binge-ED) demonstrated consistent inhibitory control deficits across studies, with greater impairment in response to disease-salient stimuli such as food/eating or body shape. In BN, the combination of high harm avoidance and reward sensitivity may manifest as high negative urgency [see Fischer et al. (2008) for review] and impulsive decision-making in order to escape negative affective states despite potential long-term negative consequences. Individuals with BN have also been shown to perform poorly on the Iowa Gambling Task (Boeka and Lokken, 2006; Liao et al., 2009; Brogan et al., 2010; Chan et al., 2014), suggesting impaired decision making that is driven by a preference for immediate gratification results from decreased cognitive control and increased reward sensitivity. Taken together, enhanced cognitive control may help to maintain persistent food restriction in AN, whereas reduced or dysregulated cognitive inhibitory control may contribute to binge/purge behavior in BN. How these behavioral symptoms are coded in neural circuits and contribute to consummatory behavior is less clear, but may result from altered functioning of neurocircuitry governing inhibitory control.

### Neurobiological evidence of altered inhibitory cognitive control in ED

fMRI studies suggest that individuals with AN have enhanced higher-order inhibitory cognitive control and individuals with BN have reduced inhibition. Recent fMRI studies of individuals with AN have focused on set-shifting, delay discounting, error monitoring, and motor inhibition to assess neural substrates of cognitive control. In general, they reveal increased activity within dorsolateral cognitive circuitry associated with set-shifting and decision-making, and reduced medial and lateral prefrontal activation during error monitoring and motor inhibitory control. For example, ill AN adults performing a set-shifting paradigm during fMRI showed greater activation of dorsolateral frontoparietal networks during task shift trials, which is thought to be indicative of excessive effortful and supervisory cognitive control (Zastrow et al., 2009). We recently showed that women remitted from AN also had elevated brain response in cognitive circuitry including the DLPFC during delay discounting, and this response was independent of hunger state (Wierenga et al., 2014a). Our findings may provide further support for enhanced cognitive control in AN, and suggest that these individuals may rely on cognitive evaluation to compensate for impaired reward processing when evaluating choices or making decisions. Conversely, ill AN adults showed reduced dACC response to commission errors on a flanker task (Pieters et al., 2007), and blunted cingulate function in relation to executive function (Ferro et al., 2005). Ill AN adults also showed reduced error monitoring demonstrated by reduced EEG error-related negativity in the context of improved performance (Pieters et al., 2007), which suggests that hypoactivity of the ACC does not necessarily lead to diminished task performance, and could indicate improved efficiency of this circuitry. A combined group of ill AN restricting and binge/purge subtypes showed decreased ventrolateral PFC activation during set-shifting error feedback trials of the Wisconsin Card Sorting Test, indicating altered response to errors when shifting cognitive set (Sato et al., 2013). Using a motor inhibition stop signal task, we showed that ill AN adolescents (Wierenga et al., 2014b) and REC AN women (Oberndorfer et al., 2011) have decreased task-related activation in the middle frontal gyrus as inhibitory demand increased (e.g., on hard trials), suggesting AN may require fewer inhibitory resources to maintain behavioral performance (Petersen et al., 1998). Taken together, results point to the possibility that AN have dysregulated information processing, with increased traffic in neurocircuits concerned with planning and consequences and more efficient processing in regions supporting conflict monitoring and motor inhibition.

Less is known about the neural mechanisms of inhibitory control in BN, though evidence suggests BN individuals may have reduced inhibition or greater dysregulation due to failure to appropriately engage frontostriatal circuits. For example, fMRI studies of the Simon Spatial Incompatibility task (Marsh et al., 2009, 2011), which requires inhibiting a more automatic response in favor of a task-relevant response, show that ill BN adults fail to appropriately activate the left inferolateral PFC, bilateral inferior frontal gyrus, lenticular and caudate nuclei, and ACC during correct responses on incongruent trials. Reduced frontostriatal response during incongruent trials was correlated with faster response times on conflict trials and greater errors across all trials in BN, suggesting that reduced activation may contribute to impulsivity and difficulties inhibiting behavior in BN. Ill BN adults also demonstrated aberrant function of the dACC, activating it slightly more when making errors than when responding correctly, an opposite pattern to control participants, suggesting that BN adults may have enhanced error detection but limited success in correcting errors. A similar study in ill BN adolescents also revealed decreased brain response during correct responding on incongruent trials in frontrostriatal circuitry, including the right inferior frontal gyrus, DLPFC and putamen, and extending to the posterior cingulate, supporting the role of deficient regulatory control in BN (Marsh et al., 2011). An additional study using a Go/No-Go task to examine inhibitory motor control revealed that ill binge-eating/purging adolescents (BN and binge-purge AN) showed increased activation in the right ACC, right DLPFC, right middle/superior temporal gyrus, precentral gyri, and hypothalamus compared to controls during correct motor response inhibition trials (Lock et al., 2011). Increased activation in the DLPFC and ACC was interpreted as inefficient or compensatory brain response related to monitoring response conflict. Cross-study differences in increased versus decreased frontostriatal activation may result from task differences and difficulty (e.g., spatial conflict resolution versus motor response inhibition), behavioral performance differences (e.g., ill adolescents did not differ from controls but adults did), group diagnosis (e.g., BN versus binge-purge behavior) or other study differences. However, taken together preliminary findings suggest dysfunctional frontostriatal neurocircuitry may contribute to dysregulated inhibitory control in BN.

Together these fMRI studies raise the intriguing hypothesis that individuals with AN and BN have altered activity of dorsal cognitive circuitry, consistent with the possibility that AN is characterized by enhanced higher-order inhibitory function and individuals with BN have reduced inhibition. Specifically, the ability to inhibit consummatory drives may be associated with neural processes underlying extraordinary self-control (e.g., exaggerated dorsal cognitive circuit function), as well as clinical symptoms such as risk avoidance and sensitivity to punishment, whereas lowered ability to self-regulate and control impulses may be associated with reduced dorsal cognitive circuit function/exaggerated ventral-striatal reward circuit function and clinical symptoms such as novelty seeking, behavioral approach, and SR that may increase one’s vulnerability to overeat. However, more studies examining *cognitive* rather than motor inhibitory control are needed to clarify the specific aspect(s) of cognitive control implicated. Of note, these conclusions rest on the notion that increased brain response reflects greater effort or recruitment of neural resources (Suskauer et al., 2008) to perform a task, and decreased brain response corresponds to more efficient processing, based on findings that brain response decreases with practice (Wartenburger et al., 2009). However, it is also possible that increased brain response reflects inefficient processing or dedifferentiation of functional specificity, and decreased brain response indicates a failure to recruit necessary resources. Thus without direct associations between brain response and behavioral outcome, and given the relatively small, but growing, repertoire of experimental tasks applied to AN and BN, the ability to draw conclusions about the mechanisms of interest based on existing neuroimaging paradigms remains limited.

## State and Trait-Related Alterations

Differentiating trait-related and state-related behavioral and neurobiological alterations in ED may be helpful in understanding the etiology and course of illness in AN and BN (Kaye et al., 2009). We reviewed earlier the evidence of childhood premorbid, genetically determined trait alterations, such as harm avoidance, behavioral inhibition, and altered response to reward (Fassino et al., 2004; Klump et al., 2004; Cassin and von Ranson, 2005; Wagner et al., 2006; Harrison et al., 2010; Lilenfeld, 2011) that contribute to a vulnerability to develop an ED and persist after recovery. Studies suggest that these traits are heritable, can be present in family members without ED and are independent of body weight (Bulik et al., 2007). This provides further evidence that these characteristics predate the disorder and may confer liability to the development of ED. Similarities across studies of ill and recovered patients support the notion that neurobiological underpinnings reflect heritable traits that contribute to the anorectic or bulimic phenotype and a vulnerability for pathological eating that persists even after nutrition and weight normalize. However, a purely trait-centric perspective assumes a stabilized system, which does not account for the relatively common transition from one ED subtype to another during the course of illness. Thus, state effects that may amplify or exaggerate trait phenomena toward one behavioral subtype or another must also be taken into account. This interaction of trait and state influences is supported by evidence that malnutrition-associated alterations exaggerate emotional dysregulation, consistent with high prevalence of co-morbid major depression, obsessive-compulsive disorder (OCD), or other anxiety disorders (Kaye et al., 2004; Godart et al., 2007) in AN. Furthermore, starvation, emaciation, and binge/purge behavior all have profound effects on the functioning of the brain and other organ systems. They cause brain volume reductions, neurochemical disturbances, and endocrine and metabolic changes (Boyar et al., 1974; Katzman et al., 1996; Pollice et al., 1997; Kaye et al., 2006) that are likely to underlie alterations in mood, cognitive function, impulse control, and autonomic and hormonal systems (Jimerson and Wolfe, 2006), that can sustain ED behaviors associated with the ill state. The fact that such disturbances tend to normalize after weight restoration or recovery suggests that they may be a consequence rather than a cause of illness.

A challenge to brain imaging studies in ill ED patients is that it is unclear whether observed changes are the cause or the result of eating pathology, and a complication of studying recovered individuals is concern that findings reflect scars or persistent consequences of the illness. Studies in animals raise the question of whether extremes of food ingestion produce chronic effects on the reward system (Johnson and Kenny, 2010; Avena et al., 2012) that may alter eating behavior. However, recent findings suggest that temperament traits, such as a trait for impulsivity, precedes, and confers vulnerability for food addiction-like behavior (Velázquez-Sánchez et al., 2014). Moreover, given the frequency of dieting and attempted weight loss in our culture, if restricting food intake produced powerful brain changes, the incidence of AN and BN would be much higher and recidivism from dieting would be lower. Taken together, data suggest EDs may be caught in a vicious cycle characterized by altered reward sensitivity and inhibitory control that dysregulates food intake, and that this in turn sensitizes/desensitizes the reward pathways in the brain to perpetuate the disordered eating behavior. However, the directionality and starting point of this pattern of behavior is difficult to resolve in the absence of data in premorbid EDs.

## Clinical Implications

EDs are chronic, relapsing illnesses (Herzog et al., 1992; Keel et al., 1999; Klein and Walsh, 2003) with a substantial medical morbidity (McKenzie and Joyce, 1992) and high mortality (Sullivan, 1995). Due to the impervious nature of ED, furthering our understanding of the temperament and neurobiological factors that contribute to the development and maintenance of disordered eating is critical to improving treatment approaches. Clinically, in AN, the bias toward overactive cognitive control circuitry and underactive reward circuitry likely interferes with motivation for treatment and ability to learn from experience. The altered response to feedback in regions associated with cognitive control and habit performance is supportive of clinical observation of enhanced inhibitory control and difficulty in changing behavior (Tchanturia et al., 2012). Ill AN individuals are highly sensitive to criticism, and are not able to appropriately assess rewarding or punishing aspects of experience in order to learn from it. Alternatively, in BN, a dysregulated response to reward and reduced inhibitory control may manifest in erratic and impulsive behaviors common in BN (e.g., substance abuse) (Gadalla and Piran, 2007; Calero-Elvira et al., 2009). Difficulty gaging the level of expected reward or punishment may also contribute to limited ability to inhibit responses when presented with appetitive stimuli. We expect that developing strategies that incorporate an understanding of altered reward sensitivity into behavioral contingency management, and that modulate anticipatory response to salient stimuli, are likely to improve treatment compliance and outcome by enhancing motivation and reducing uncertainty.

## Conclusion

EDs may be better understood based on a continuum of interacting neurocognitive constructs underlying consummatory behavior. Behavioral, cognitive, and neuroimaging evidence suggests an altered balance of reward and inhibition can explain disordered food consumption in ED, which corresponds to disruption of both ventral limbic reward circuitry and dorsal cognitive circuitry. For example, restricted eating in AN may emerge from excessive inhibition and diminished valuation of reward. In contrast, the combination of dysregulated inhibitory control and excessive reward sensitivity may lead to a pattern of under- and over-consumption characteristic of AN-BN and BN. While considerable research in AN supports this model, continued research investigating the contribution of reward and inhibition to BN symptomatology is needed. The lack of a mechanistic understanding of ED has thwarted efforts to develop evidence-based treatment approaches. Viewing eating pathology from the perspective of an altered balance of inhibitory control and reward processing that is driven by changes in neural circuitry supporting these constructs provides a foundation for developing more specific and effective interventions for these debilitating and deadly disorders.

## Conflict of Interest Statement

The authors declare that the research was conducted in the absence of any commercial or financial relationships that could be construed as a potential conflict of interest.
